# Molecular characterization and phylogenetic study of African swine fever virus isolates from recent outbreaks in Uganda (2010–2013)

**DOI:** 10.1186/1743-422X-10-247

**Published:** 2013-08-01

**Authors:** David Kalenzi Atuhaire, Mathias Afayoa, Sylvester Ochwo, Savannah Mwesigwa, Julius Boniface Okuni, William Olaho-Mukani, Lonzy Ojok

**Affiliations:** 1 College of Veterinary Medicine, Animal resources and Biosecurity, Makerere University, P.O. BOX 7062, Kampala, Uganda; 2 National Agricultural Research Organization, National Livestock Resources Research Institute, P.O. BOX 96, Tororo, Uganda; 3 African Union-Interafrican Bureau of Animal Resources, P.O. BOX 30786, Nairobi, Kenya

**Keywords:** African swine fever virus, Genotyping, P54, p72, pB602L (central variable region) sequencing

## Abstract

**Background:**

African swine fever (ASF) is a highly lethal and economically significant disease of domestic pigs in Eastern Africa particularly in Uganda where outbreaks regularly occur. Sequence analysis of variable genome regions have been extensively used for molecular epidemiological studies of African swine fever virus (ASFV) isolates. By combining p72, P54 and pB602L (CVR), a high level resolution approach is achieved for viral discrimination. The major aim of this study therefore, was to investigate the genetic relatedness of ASF outbreaks that occurred between 2010 and 2013 in Uganda to contribute to the clarification of the epidemiological situation over a four year period.

**Methods:**

Tissue samples from infected domestic pigs associated with an ASF outbreak from 15 districts in Uganda were confirmed as being infected with ASFV using a p72 gene-based polymerase chain reaction amplification (PCR) assay recommended by OIE. The analysis was conducted by genotyping based on sequence data from three single copy ASFV genes. The E183L gene encoding the structural protein P54 and part of the gene encoding the p72 protein was used to delineate genotypes. Intra-genotypic resolution of viral relationships was achieved by analysis of tetramer amino acid repeats within the hypervariable CVR of the B602L gene.

**Results:**

Twenty one (21) ASF outbreaks were confirmed by the p72 ASF diagnostic PCR, however; only 17 isolates were successfully aligned after sequencing. Our entire isolates cluster with previous ASF viruses in genotype IX isolated in Uganda and Kenya using p72 and P54 genes. Analysis of the CVR gene generated three sub-groups one with 23 tetrameric amino acid repeats (TRS) with an additional CAST sequence, the second with 22 TRS while one isolate Ug13. Kampala1 had 13 TRS.

**Conclusion:**

We identified two new CVR subgroups different from previous studies. This study constitutes the first detailed assessment of the molecular epidemiology of ASFV in domestic pigs in the different regions of Uganda.

## Background

African swine fever virus (ASFV) is a large double stranded DNA virus with 170–190 kb, classified as sole member of *Asfarviridae* family [1]. It is responsible for a highly contagious and fatal disease of domestic pigs, representing a serious threat to swine industry in East Africa and the rest of the world. Though first isolated in domestic pigs in 1921 in Kenya, the virus occurs naturally in both vertebrate and invertebrate sylvatic hosts throughout sub-Saharan Africa and is transmitted to domestic pigs when infected ticks of the *Ornithodoros moubata* complex feed on them [2]. A domestic pig cycle, which is apparently not reliant on the presence of the tick vector, is believed to occur in both West and East Africa [2]. ASF is endemic in most of sub-Saharan Africa, including the island of Madagascar; the highest incidence of disease being recorded from the equator to the northern Transvaal in southern Africa. Disease outbreaks have also occurred in Europe, South America and the Caribbean. In 2007, it was introduced into Georgia, most probably through infected pig-meat that was unloaded from a ship, recycled and fed to local pigs and has since spread throughout the Caucasus and into southern Russia [3].

Pig farming is one of the fastest growing livestock activities in the rural areas of Uganda and has become very attractive throughout the country as a means of increasing food, income and employment but has on several occasions been hampered by ASF. According to reports, Uganda has the largest and fastest growing pig production in Eastern Africa with the pig population standing at 3.2 million [4]. But ASF is an economically important and frequently lethal disease of domestic pigs which has hampered the development. Thus, the outbreaks of ASFV are still a great challenge for the swine industry in Uganda. At present there is no treatment or vaccine available, and control is based on rapid laboratory diagnosis and the enforcement of strict sanitary measures [5].

Different epidemiological regions and outbreaks are known to exhibit different virus population dynamics, degrees of diversity and different disease manifestations in susceptible hosts. Genotyping of ASFV isolates is vital in establishing the patterns of outbreaks for future control and eradication of the disease. Sequence analysis of variable genome regions has been extensively used for molecular epidemiological studies of ASFV isolates [6-11] and in Uganda [12]. A combined p72–CVR approach has been successfully used to investigate the field heterogeneity of viruses causing recent and historical outbreaks in Eastern and Southern Africa [7-9]. Previous studies have demonstrated the value of full P54 gene sequencing for providing additional, intermediate resolution when typing of ASFV viruses [13]. By combining p72, P54 and pB602L (CVR), a high level resolution approach is achieved for viral discrimination [7,14].

The major aim of this study therefore, was to investigate the genetic relatedness of ASFV outbreaks that occurred between 2010 and February 2013 in Uganda to contribute to the clarification of the epidemiological situation over a four year period. For this purpose we applied genotyping to a wide range isolates collected from outbreaks in domestic pigs based on partial sequencing of p72, full P54 gene sequencing, and sequencing of the CVR of the B602L gene. This study constitutes the first detailed assessment of the molecular epidemiology of ASFV in domestic pigs in the different regions of Uganda.

## Results

### ASF diagnosis

**Figure 1:**
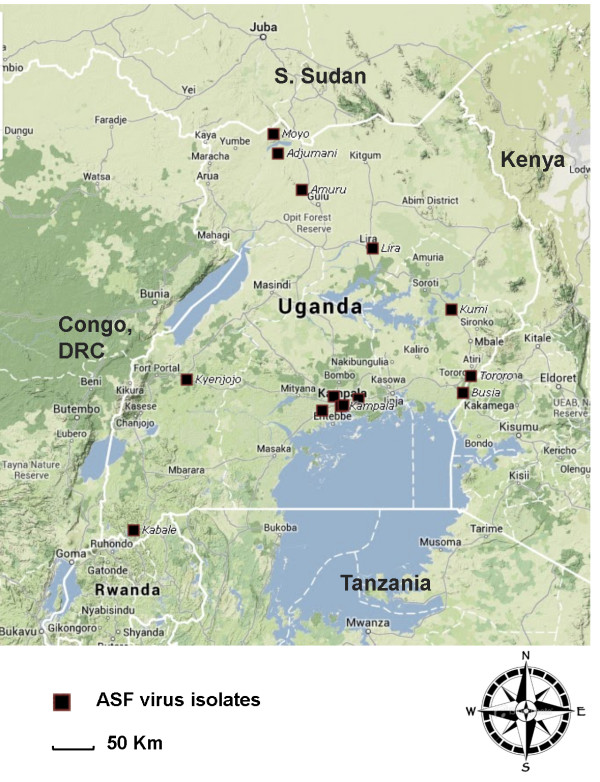
Map of Uganda showing the origins of African swine fever virus isolates obtained in this study.

A total of 30 outbreaks were reported during the study duration (2011–2013) as a result of reports of pig deaths in the different areas in Uganda. Out of the 30 outbreaks, 21 tissue samples tested positive with the OIE diagnostic PCR. The origins of ASFV isolated in this study are shown in Figure 1.

### p72 gene phylogeny

In order to classify the Ugandan isolates characterized in this study into one of the known p72 genotypes in East Africa [15], the C-terminal end of the p72 gene was amplified and sequenced. Sequences obtained were compared with 42 of the sequences obtained in GenBank comprising representatives of genotypes IX, X, VIII, and II (see Additional file 1).

**Figure 2:**
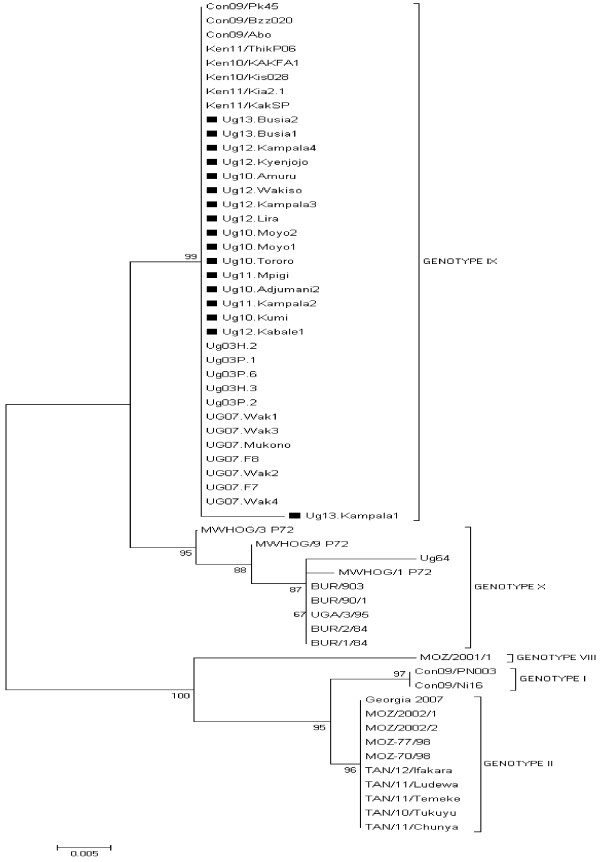
**Evolutionary relationships of p72 genotypes: neighbor-joining tree of the p72 gene.** The analysis involved 59 nucleotide sequences. The p72 sequences from this study are marked with ■.

The analysis of the p72 partial gene sequences from each of the 17 ASFV isolates showed that they were identical at the nucleotide level. The phylogenetic analysis established that all the Ugandan viruses obtained in this study were placed in the p72 genotype IX together with some viruses isolated in previous studies in Uganda, Kenya, and Congo whereas isolates from Burundi, one from Uganda (Ug64), Mozambique and Georgia in Europe clustered in other genotypes as shown in Figure 2. The GenBank accession numbers of the p72 ASFV genotypes isolated in this study are shown (see Additional file 2).

### P54 gene phylogeny

**Figure 3:**
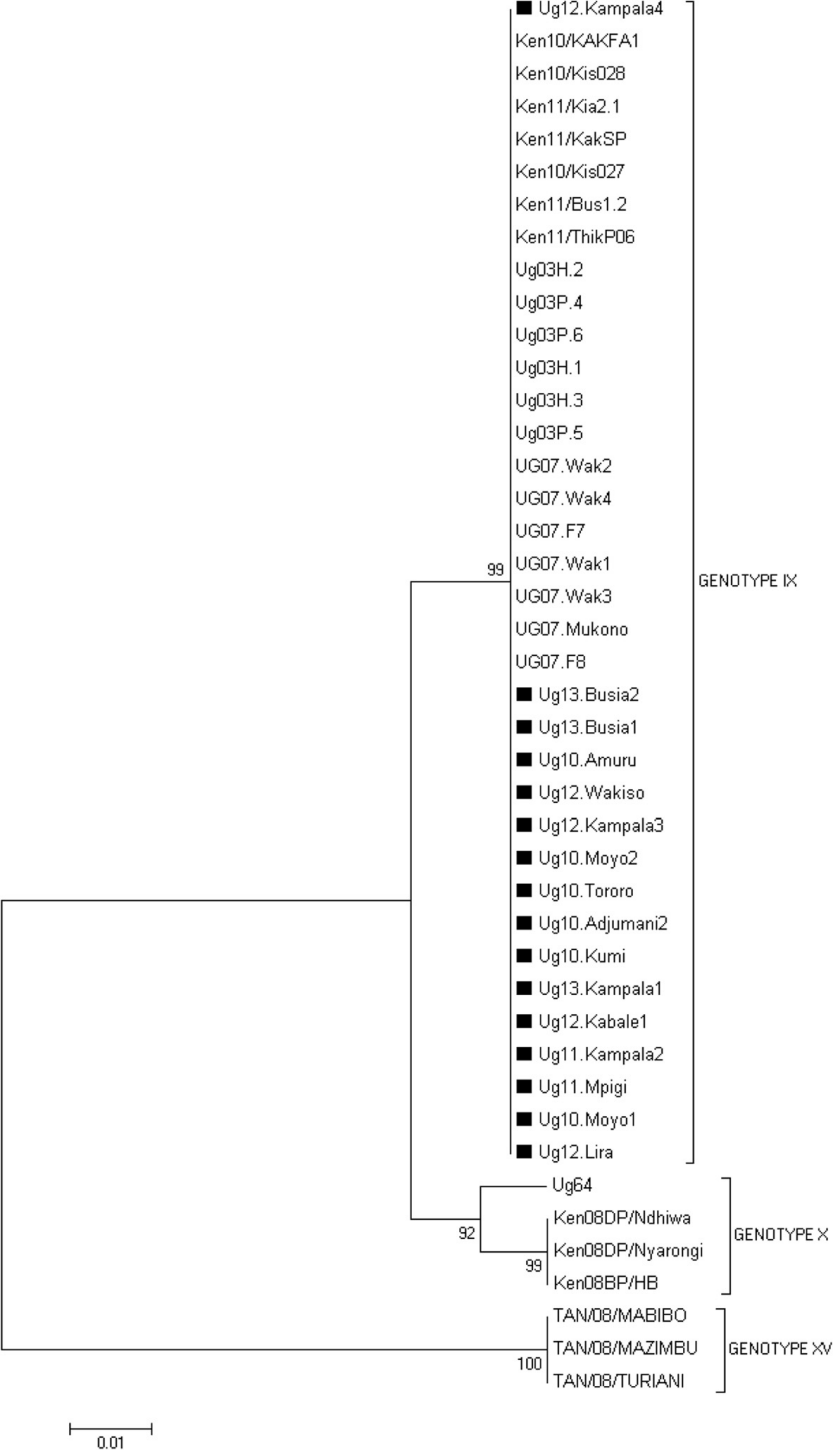
**Evolutionary relationships of P54 genotypes: the neighbor-joining tree of the P54 gene.** The analysis involved 43 nucleotide sequences. The P54 sequences from this study are marked with ■.

Previous studies have confirmed P54 sequencing as a valuable additional genotyping method for molecular epidemiological studies of genotype IX ASF viruses [6,8,14]. PCR amplification of the fragment containing the complete P54 gene from all of the Ugandan isolates in this study produced products of approximately 676 bp. The nucleotide sequence analysis of the P54 gene showed that all the isolates were identical. The sequences of the 16 Ugandan isolates were compared with 27 P54 ASFV sequences retrieved from the GenBank (see Additional file 1). The phylogeny revealed that the Ugandan viruses obtained in this study cluster with the majority of the viruses from previous outbreaks in Uganda and Kenya (Figure 3). The GenBank accession numbers of the P54 ASFV genotypes isolated in this study are shown (see Additional file 2).

### Intra-genotypic resolution (CVR) of homogenous p72 genotype IX Ugandan isolates

In order to delineate the p72 genotype IX obtained in this study at a higher resolution the CVR of the B602L gene was analyzed. Amplification of the CVR gave products of varying sizes (400–600 bp); with Ug13Kampala1 isolate showing a band smaller than other isolates of approximately 400 bp. The Ugandan isolates characterized in this study clustered with isolates from previous studies in Uganda and some from Kenya. However, differences were mainly observed in the number of tetrameric amino acid repeats of the isolates in this study. Isolates Ug13.Kampala1, Ug12.Kampala4, Ug11.Mpigi, Ug12.Wakiso, Ug10.Adjumani2, Ug10. Moyo1, Ug12. Lira, Ug10. Amuru and Ug12. Kabale had a change due to the presence of a single internally located tetrameric repeat (CAST). One of the isolates Ug13.Kampala1 was quite different from the rest due to the absence of 11 tetrameric amino acid repeats (Table 1) suggesting a different CVR subgroup. Isolates Ug11.Kampala2, Ug12.Kampala3, Ug10.Namasuba, Ug10.Tororo, Ug13.Busia1, Ug13.Busia2, Ug10.Kumi, Ug10.Moyo2 and Ug12.Kyenjojo were identical in the tetrameric amino acid sequences to isolates from a 2003 Kenyan outbreak but were different from isolates causing an outbreak in Uganda in 2007 which lacked a CADT tetrameric repeat (Table 1) [12].

**Table 1:** Amino acid sequence of the tetrameric repeats that constitute the CVR of the B602L gene identified in viruses belonging to p72 genotype IX

**Isolat**	**Country**	**p72 genotype**	**CVR amino acid sequence**	**No. of repeats**	**CVR enBank accession no.**	**Reference**
Ug13. Kampala1	Uganda	IX	AAAA--A----------BNABNaBA	13	KC990856	This study
Ug12. Kampala4	Uganda	IX	AAAABNABBNABB-----aaBBNABNaBA	24	KC990870	This study
Ug11. Mpigi	Uganda	IX	AAAABNABBNABB-----aaBBNABNaBA	24	KC990861	This study
Ug12. Wakiso	Uganda	IX	AAAABNABBNABB-----aaBBNABNaBA	24	KC990867	This study
Ug10. Adjumani2	Uganda	IX	AAAABNABBNABB-----aaBBNABNaBA	24	KC990860	This study
Ug10. Moyo1	Uganda	IX	AAAABNABBNABB-----aaBBNABNaBA	24	KC990863	This study
Ug12. Lira	Uganda	IX	AAAABNABBNABB-----aaBBNABNaBA	24	KC990865	This study
Ug10. Amuru	Uganda	IX	AAAABNABBNABB-----aaBBNABNaBA	24	KC990868	This study
Ug12. Kabale1	Uganda	IX	AAAABNABBNABB-----aaBBNABNaBA	24	KC990857	This study
Ug11. Kampala2	Uganda	IX	-AAABNABBNABB------aaBBNABNaBA	23	KC990859	This study
Ug12. Kampala3	Uganda	IX	-AAABNABBNABB------aaBBNABNaBA	23	KC990866	This study
Ug10. Namasuba	Uganda	IX	-AAABNABBNABB------aaBBNABNaBA	23	KC990873	This study
Ug10. Tororo	Uganda	IX	-AAABNABBNABB------aaBBNABNaBA	23	KC990862	This study
Ug13. Busia1	Uganda	IX	-AAABNABBNABB------aaBBNABNaBA	23	KC990871	This study
Ug13. Busia2	Uganda	IX	-AAABNABBNABB------aaBBNABNaBA	23	KC990872	This study
Ug10. Kumi	Uganda	IX	-AAABNABBNABB------aaBBNABNaBA	23	KC990858	This study
Ug10. Moyo2	Uganda	IX	-AAABNABBNABB------aaBBNABNaBA	23	KC990864	This study
Ug12. Kyenjojo	Uganda	IX	-AAABNABBNABB------aaBBNABNaBA	23	KC990869	This study
Ken11/KakSP	Uganda	IX	-AAABNABBNABB------aaBBNABNaBA	23	AGC93414.1	[11]
Ken11/Kia2.1	Kenya	IX	-AAABNABBNABB------aaBBNABNaBA	23	AGC93412.1	[11]
Ken10/Kis028	Kenya	IX	-AAABNABBNABB------aaBBNABNaBA	23	AGC93410.1	[11]
Ken10/KAKFA1	Kenya	IX	-AAABNABBNABB------aaBBNABNaBA	23	AGC93408.1	[11]
Ken11/ThikP06	Kenya	IX	-AAABNABBNABB------aaBBNABNaBA	23	AGC93413.1	[11]
Ken11/Bus1.2	Kenya	IX	-AAABNABBNABB------aaBBNABNaBA	23	AGC93411.1	[11]
Ken10/Kis027	Kenya	IX	-AAABNABBNABB------aaBBNABNaBA	23	AGC93409.1	[11]
UG07. Wak2	Uganda	IX	-AAABNABBNABB-------aa-BNABNaBA	22	ACZ18202.1	[12]
UG07. Wak4	Uganda	IX	-AAABNABBNABB-------aa-BNABNaBA	22	ACZ18204.1	[12]
UG07.F7	Uganda	IX	-AAABNABBNABB-------aa-BNABNaBA	22	ACZ18206.1	[12]
UG07. Wak1	Uganda	IX	-AAABNABBNABB-------aa-BNABNaBA	22	ACZ18201.1	[12]
UG07. Mukono	Uganda	IX	-AAABNABBNABB-------aa-BNABNaBA	22	ACZ18205.1	[12]
UG07.F8	Uganda	IX	-AAABNABBNABB-------aa-BNABNaBA	22	ACZ18207.1	[12]
Uga_95/1	Uganda	IX	AAABNABBNABBNABBaaBBNABNaBA	27	CAJ90783.1	[16]

Key: A, CAST; a, CVST; B, CADT, CADI; N, NVDT. Dashes indicate gaps introduced manually to enable similarities between sequences to be easily visible. The number following the name of the virus isolate indicates the isolation year.

## Discussion

ASF is endemic in Uganda [17] and several outbreaks have been reported in the country since 2001 [18] and continues to impact on the farmers economically. The inability of ASFV to induce neutralizing antibodies has continuously hampered the prevention and control of the disease by vaccination in endemic areas. The formulation of appropriate disease control strategies requires intensive molecular epidemiological investigations that would benefit greatly from insights provided by previous and recent ASF outbreaks in Uganda and the neighbouring countries. The present study aimed at characterizing the ASF viruses causing disease outbreaks in Uganda between 2010 and February 2013 based on the partial end of the C terminal p72 gene, full length P54 gene and the CVR of the B602lL gene as previously described [12].

In this study, the OIE recommended PCR detected 21 of the 30 reported outbreaks in Uganda. Since PCR is extremely sensitive it is possible that the nine outbreaks (reports of pig deaths) could have been caused by a different aetiology, since clinical signs of ASF are not pathognomonic. This shows that there are sometimes false alarms of ASF outbreaks in the country and that confirmatory laboratory diagnosis should be done before control measures are instituted.

Phylogenetic analysis based on the p72 gene grouped all the Ugandan isolates from this study into genotype IX. Our findings agree with a previous study of ASFV isolates causing outbreaks in Uganda that placed them in genotype IX [12]. Our isolates also cluster with isolates causing outbreaks in Congo in 2009 and in Kenya 2010 and 2011 suggesting that this same genotype is circulating in the three countries. A recent study has assessed the role of cross border pig movements and related them to occurrence of ASF outbreaks in Ugandan districts adjacent to international borders [18]. Analysis of the full length P54 gene produced similar results obtained using the p72 gene with the current Ugandan isolates clustering with isolates from 2003 and 2007 outbreaks in Uganda, and outbreaks occurring in Kenya between 2010 and 2011 [12].

Though p72 and P54 genes are useful for identifying the major ASFV genotypes, higher discrimination of virus isolates enables more detailed dissection of the genotypes for epidemiological analysis and classification. Intra-genotyping resolution by the CVR has been shown to be beneficial for identifying epidemiological links between different outbreaks caused by genotype VIII viruses and is therefore advocated for clarifying viral relationships within homogenous p72 genotypes that are associated with the domestic pig cycles [19]. Therefore, sequences of the CVR characterized by the presence of tetrameric amino acid repeats were generated from the Ugandan isolates. Three different CVR variants were observed based on the number of tetrameric amino acid repeat sequences (TRS). Isolate Ug13.Kampala1 had 13 TRS meaning it lacked over 11 TRS. Isolates Ug12.Kampala4, Ug11.Mpigi, Ug12.Wakiso, Ug10.Adjumani2, Ug10.Moyo1, Ug12. Lira, Ug10.Amuru and Ug12 had 24 TRS. The third subgroup of the Ugandan isolates Ug11. Kampala2, Ug12.Kampala3, Ug10.Namasuba, Ug10.Tororo, Ug13.Busia1, Ug13.Busia2, Ug10.Kumi, Ug10.Moyo2 and Ug12.Kyenjojo had 23 TRS and these isolates were identical to viruses causing outbreaks in Kenya in 2010 and 2011 suggesting that the same virus is circulating between the two countries. The presence of a domestic pig-associated genotype IX causing ASF outbreaks in both Kenya and Uganda and evidence of trans-boundary transmission between these two countries indicates that a regional approach to ASF control would be more efficient. Interestingly, our isolates based on CVR analysis were different from those identified in the 2007 Ugandan outbreaks due to the presence of an extra TRS (CADT). This could be as a result of a minor mutation of the virus circulating in Uganda.

## Conclusion

This is the first study detailing the molecular epidemiology of ASFV from reported outbreaks in domestic pigs from Uganda. Findings suggest that one p72 genotype IX virus is circulating in Uganda and that the same virus has caused outbreaks in Kenya in 2010 and 2011. Three subgroups of ASF viruses were identified based on analysis of the CVR gene. Therefore characterization of viruses causing outbreaks in Uganda and neighbouring countries needs to be carried out on a regular basis in order to ascertain the changes in genotypes and their origins. Additionally detailed studies need to be carried out on ASF virus isolates from the sylvatic cycle in order to assess the role of wild swine and *Ornithodorus* ticks in the epidemiology of ASF in Uganda.

## Methods

### Study area and virus isolates

Samples (blood and tissues) were collected from pigs in the districts that reported death of pigs in the different regions of Uganda. The pigs were suspected to have died as a result of ASF because of the massive deaths and postmortem findings. A total of 30 samples collected in 30 outbreaks were subjected to an ASF diagnostic PCR out of which 21 tested positive. Virus isolation (VI) was performed using homogenized pooled tissues from the same animal whereas; blood was used for VI for only one isolate Ug12.Kampala4. Three-day-old cultures of primary pig bone marrow cells grown in Earle’s medium supplemented with 10% pig serum were used to isolate virus from only the samples that tested positive with diagnostic PCR [20]. Twenty one cell-culture isolates derived from outbreaks in domestic pigs in Uganda (2010–2013) were selected for the p72 sequencing, P54 sequencing and CVR characterization components of this study.

### DNA extraction and genomic amplification

Viral DNA was extracted directly from 200 μl aliquots of blood collected in EDTA tubes or of tissue sample homogenates and virus cultures by using a DNeasy Blood and tissue kit (Qiagen® USA). A 278 bp region corresponding to the central portion of the p72 gene was amplified using the diagnostic primers, primer 1 (5′-ATGGATACCGAGGGAATAGC-3′) and primer 2 (5′-CTTACCGATGAAAATGATAC-3′) to confirm the presence of ASFV DNA [14].

Epidemiological primers which amplify the C-terminal region of the p72 gene (478 bp), p72-U (5′-GGCACAAGTTCGGACATGT-3′) and p72-D (5′-GTACTGTAACGCAGCACAG-3′) as described previously were used for p72 genotyping [6]. The complete gene encoding the P54 protein was amplified using the primers PPA722 (5′-CGAAGTGCATGTAATAAACGTC-3′) and PPA89 (5′-TGTAATTTCATTGCGCCACAAC-3′) flanking a 676 bp DNA fragment [13]. The CVR located in the B602L gene was amplified using the primer pairs CVR-FL1 (5′-TCGGCCTGAAGCTCATTAG-3′) and CVR-FL2 (5′-CAGGAAACTAATGATGTTCC-3′) flanking a variable in size DNA fragment [7]. Conditions for the three PCR assays were as previously described [13] though the annealing temperature was reduced to 50°C.

### Nucleotide sequencing and analysis

Amplification products of the expected size were identified against a molecular weight marker, following electrophoresis on a 2% agarose gel. Bands of correct size were excised and purified by means of a Ron’s Gel Extraction Kit (BIORON®, Germany) according to manufacturer specifications and sent to Macrogen Europe for sequencing. The sequences obtained were submitted to BLAST (Basic Local Alignment Search Tool) [8] in order to identify their similarity with sequences obtained from the GenBank. Sequence alignment was performed using CLUSTAL W [9] with manual adjustments. For phylogenetic analysis, the MEGA 5.0 program [10] was used to construct neighbour-joining (NJ) trees with 1000 bootstrap replications. The trees were drawn to scale, with branch lengths in the same units as those of the evolutionary distances used to infer the phylogenetic trees. The evolutionary distances were computed using the p-distance method and are in the units of the number of base differences per site. Codon positions included were 1st + 2nd + 3rd + Noncoding. All ambiguous positions were removed for each sequence pair. There were a total of 393 and 597 positions in the final p72 and P54 datasets.

## Abbreviations

ASF: African swine fever; ASFV: African swine fever virus; CVR: Central variable region; PCR: Polymerase chain reaction; OIE: Office International des Epizooties; TRS: Tetrameric amino acid repeat sequence.

## Competing interests

The authors declare that they have no competing interests.

## Authors’ contributions

DKA contributed to the conception of the idea, design, data collection, drafting and writing of the manuscript. MA contributed to data collection and culturing of the viruses. SO contributed to the laboratory work and drafting of the manuscript. SM contributed to data analysis and writing of the manuscript. JOB contributed to conception of the idea, data collection and writing of the manuscript. WO contributed to conception and writing of the manuscript. LO contributed to conception of the idea, design and writing of the manuscript. All read and approved the manuscript.

## Supplementary Material

Additional file 1 **Summary of African swine fever virus isolates from previous studies used in phylogenetic analysis.** The file contains a table with African swine fever virus isolates obtained from Data bases, with accession numbers, genotypes and their reference. These were used for phylogenetic analysis with the isolates obtained in this study. Click here for file

Additional file 2 **Accession numbers of sequences used to characterize ASFV isolates obtained in this study.** The file contains a table with African swine fever virus isolates obtained from this study, year of outbreak, p72 and P54 genotype accession numbers, and the district where ASF outbreak occurred. Click here for file
